# Facial nerve grafting and end-to-end anastomosis in the middle ear - tympanic cavity and mastoid

**DOI:** 10.5935/1808-8694.20130079

**Published:** 2015-10-08

**Authors:** Nelson Álvares Cruz Filho, José Evandro Prudente de Aquino, Luís Francisco de Oliveira

**Affiliations:** a PhD in Otorhinolaryngology, School of Medicine of the University of São Paulo (Head of Otology, Beneficiência Portuguesa Hospital in São Paulo, Ivan F. Barbosa Clinic).; b PhD in Otorhinolaryngology, Paulista School of Medicine, Federal University of São Paulo (Head of ENT at the Santa Casa de Lorena).; c Former Preceptor, ENT Residency, Ivan F. Barbosa Clinic, Beneficiência Portuguesa Hospital in São Paulo (MD, ENT). Beneficiência Portuguesa Hospital in São Paulo.

**Keywords:** anastomosis, surgical, ear, middle, facial nerve, microsurgery

## Abstract

Sectioned facial nerves can be repaired with grafting or end-to-end anastomosis.

**Objective:**

To discuss these repair procedures and what can be expected of them.

**Method:**

Seven patients with sectioned facial nerves were included in the study. Four underwent grafting and three were offered end-to-end anastomosis. Facial nerve palsy was iatrogenic in five patients and was caused by bullet wounds in two. Assessment of motor function recovery was based on Janssen's scale.

**Results:**

Mean motor recovery was rated at 72.5% for subjects offered grafting and 73.3% for patients submitted to anastomosis.

**Conclusion:**

1. Grafting and anastomosis are proper solutions to repair sectioned facial nerves; complete recovery is never attained; synkinesis may occur. 2. In principle anastomosis is the procedure of choice, but when there is minimal traction in the facial nerve stump grafting is preferred. 3. Both procedures yielded mean motor recovery rates above 70% (72.5% for grafting and 73.3% for anastomosis).

## INTRODUCTION

Sectioned facial nerves can be repaired with grafting or end-to-end anastomosis. Intratemporal sectioning of the facial nerve may occur due to iatrogenesis, head trauma, or bullet wounds. The facial nerve may also be willingly sectioned in facial nerve schwannoma surgery^1^. When conditions are favorable, the facial nerve trunk should be approached during surgery to allow patients a possibility of recovering facial movements and future referral to plastic surgery.

This paper aims to discuss the approaches mentioned above and show what can be expected of them based on seven personal cases and literature reports. Very little was published in the last decade on facial nerve repair procedures. The literature features papers on other surgical approaches applied to the facial nerve which will not be discussed in this study.

## METHOD

Seven patients were operated for facial nerve repair in the last eight years in our institution. Four were males and three were females, with ages ranging from five to 54 years (mean of 28.7 years). Four grafting procedures and three end-to-end anastomoses were carried out.

All patients had symmetric normal tearing according to Schirmer's test. All individuals with intact tympanic membranes underwent pure-tone audiometry, speech discrimination, and impedance testing. Five subjects had iatrogenic facial palsy after mastoidectomy and two had bullet wounds. Six patients were referred for palsy care and in one case our team was called to aid a colleague in the final stages of surgery.

Six patients had facial nerve palsy immediately after trauma episodes. One patient was shot in the ear and was unconscious for two days, and was thus unaware of the exact time of facial nerve palsy onset. This patient's temporal bone CT scans revealed that part of the bullet was located in the tympanic segment of the facial nerve. In the other case of bullet injury, CT scans showed the firearm projectile was lodged in the mastoid segment of the nerve.

The transmastoid approach was used in all patients; five had canal-wall-up mastoidectomies and two had radical mastoidectomies. Four patients had minor middle ear effusion.

Sural nerve grafts were used in three cases and great auricular nerve graft in one patient.

**Figure 1:**
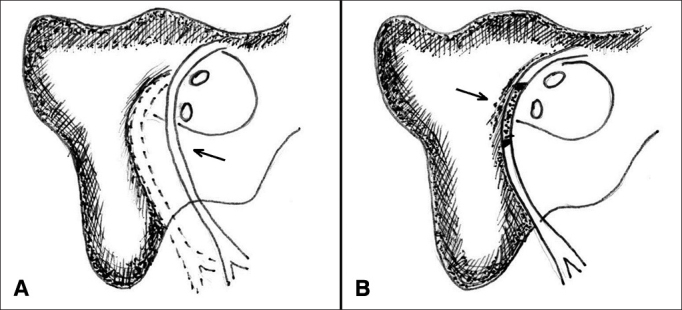
A: Facial nerve anastomosis; B: Facial nerve grafting.

In the three patients offered anastomosis, the facial nerve was removed from the canal to repair the nerve path and ensure good approximation of the nerve stumps (Figure 1).

In only two of the seven surgeries (one grafting and one anastomosis procedure) epineural sutures were made with Mononylon 10-0^®^. In the other procedures, Gelfoam^®^ and blood clot were used to hold the graft or anastomosis in the desired position. Five patients had a small portion of the stump resected to eliminate possible traumatic neuromas. This procedure was not needed in two cases as the injuries were recent. Sectioning incisions were made perpendicularly to the free ends of the facial nerve. Graft lengths of 7, 8, 11 and 12 mm were used.

The table developed by Janssen^2^ was used in data analysis, as it assesses segments of the face and reduces examiner subjectivity (Table 1).

**Table 1:** Janssen's table.

Frontal contraction	10%
Eye closure	30%
Labial commissure	30%
Rest	30%
Total	100% (normal face motricity)

The electromyograms of five patients were available five and six months after surgery, thus providing information on reinnervation of the face. Electromyography was not ordered for the pediatric patient. One patient was away for 11 months and came back with good recovery of facial movements. This study was approved by the institution's Research Ethics Committee (permit #500-09).

## RESULTS

**Figure 2:**
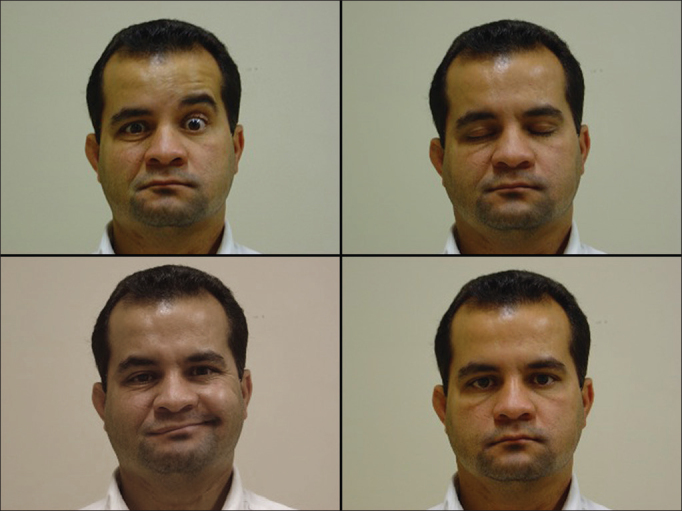
Anastomosis. Absent frontal muscle contraction to the right; complete closure of right eye; contraction of labial commissure; good facial symmetry at rest.

The tables show results for facial motricity. In the anastomosis patients, the first signs of facial muscle motion were seen six to seven months after nerve repair surgery (Figure 2 and Table 2).

**Table 2:** Facial nerve anastomosis.

	Period of palsy before surgery	Janssen's table	
		Before surgery	After surgery
1. EGS	Immediate repair	20%	70%
2. RFS	32 days	15%	75%
3. JAM	46 days	15%	75%

**Figure 3:**
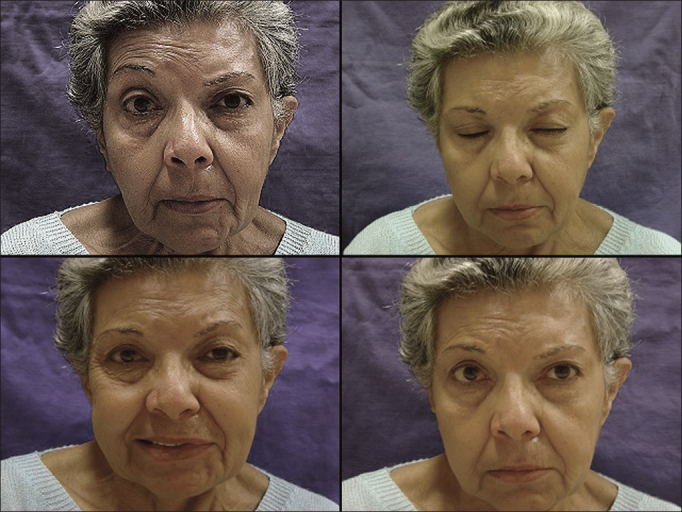
Grafting. Absent frontal muscle contraction to the left; complete closure of left eye; contraction of labial commissure; good facial symmetry at rest.

Grafting patients took longer to show signs of recovery (Figure 3 and Table 3).

**Table 3:** Facial nerve grafts.

	Period of palsy before surgery	Janssen's table	
		Before surgery	After surgery
1. VLN	4 days	15%	75%
2. MC	2 days	20%	80%
3. LCV	7 days	25%	65%
4. AMC	10 days	20%	70%

## DISCUSSION

An important principle in facial nerve surgery is to approach the nerve trunk first. However, this is possible only when clinical and electrical tests show the effector muscle is still good and the injured segment can be accessed through surgery^3^.

Despite their limitations, facial nerve grafting and anastomosis may produce partial recovery of facial movements^4^. Surgery is clearly superior to offering no treatment^5^. Given the choice between grafting and anastomosis, which is better for the patient?

Both approaches were discussed and compared in this study, vis-à-vis the personal experience of the authors and the international literature.

In cases of sectioned facial nerve, suturing is hardly possible without rerouting^3^. Rerouting consists of establishing a new route for the facial nerve, in order to approximate the stumps of the sectioned nerve. Nerve gaps of up to 23 mm^6^ can be bridged. Some of the advantages of rerouting are the non-interposition of a different nerve (graft) between the injured ends of the facial nerve, and the production of only one junction between nerve stumps. This approach has been criticized for interfering with blood supply when the nerve is immobilized^6^, ^7^. Additionally, all nerves used as graft have multiple bundles, whereas the facial nerve has one bundle only, offering better regeneration when the stumps of the nerve itself are approximated^6^.

Salaverry^6^ described seven intratemporal rerouting procedures, applied based on the affected segments of the facial nerve and the extent of the injury.

Grafts allow the facial nerve to be repaired with minimal nerve end handling^5^, thus leading to lesser impact on nerve vascularization^7^ and preserving the anatomy of the middle ear^5^. The disadvantages of grafting include the interposition of a different nerve between the stumps of the facial nerve, two junctions between the ends of the affected nerve and the graft, and loss or reduction of donor site sensitivity^8^. Grafts are more commonly made from the great auricular nerve, a branch of the superficial cervical plexus^9^, ^10^, ^11^, ^12^, ^13^, or the sural nerve, when longer grafts are required^9^, ^10^.

The sural nerve was used in three patients and the great auricular on one subject. The first was preferred for not producing scars on the neck.

The following caution measures are required when placing grafts:

1. Resection of fibrotic devitalized facial nerve stumps. Nerve stumps swell when the facial nerve is sectioned. Unless the stumps are approximated quickly, the disorganized proliferation of axons in the proximal nerve ends give rise to traumatic neuromas, which have to be removed^6^. Formation of neuromas^7^ or gliomas has not been observed within 72 hours of facial nerve injury. Retraction and stump fibrosis have been described in the facial nerves of pigs five days after injury^14^.
2. The ends of the facial nerve must be sectioned in a plane perpendicular to the axis of the nerve trunk, as described in the literature^10^, ^12^, ^13^, ^15^, ^16^, ^17^, ^18^. Fisch & Rouleau^4^ and Fisch^9^ recommended stumps and graft ends should be sectioned in an oblique plane to increase contact surface area. Cruz & Macha^12^ reported it is harder to attain perfect parallelism in oblique sectioning, which is why they prefer perpendicular sectioning. Our patients were offered sectioning of facial nerve ends in a plane perpendicular to the axis of the nerve trunk.
3. Perfect juxtaposition between the facial nerve and the graft using minimal epineural suture due to the risk of nerve fibrosis^4^, ^5^.
  The area of the fallopian canal is not subject to movement, and juxtaposition alone can be performed without the need for sutures^7^, as was the case of three of our patients offered Gelfoam^®^ and blood clots to keep the graft in position. Graft patients were given two sutures on the epineurium with Mononylon 10-0^®^.
  Suturing appears not to increase nerve junction effectiveness. Experiments with cats revealed that gluing leads to better reinnervation than epineural sutures with 10-0 line or stump approximation^19^. Some authors have successfully used fibrin glue in facial nerve surgery in a significant number of patients^5^, ^18^
  Portmann et al.^3^ described the placement of a vein graft under the nerve graft and the use of glue to attach the vein to the nerve graft off of the junction between the nerve graft and the facial nerve stumps, so that glue does not leak into the junction point.
4. Fitting the graft to the bed.
  The graft must be longer than the space between facial nerve stumps due to nerve retraction^12^. Avoid twisting the nerve or tensioning the system^12^. The graft must lie on a bed, such as the facial nerve canal, and should not be left suspended or unsupported^18^.

Author preferences vary between anastomosis and grafting. Spector et al.^8^ reported better outcomes with rerouting. They found that grafts allow for more mass movements, less individualized facial movements, and more accentuated synkinesis.

Cazelles et al.^5^ gave preference to anastomosis for nerve injuries of one to four millimeters, rerouting only one nerve end. Grafting was preferred for injuries greater than five millimeters.

McCabe^7^ preferred grafting to anastomosis, and reported that ample mobilization of the nerve was not beneficial and impacted nerve vascularization.

Sterkers et al.^18^ compared the outcomes of both approaches in 60 patients and observed better recovery in the group submitted to grafting. Grafting was chosen in cases of minimal stump tensioning, as also observed in our study.

How long can you wait before surgery?

In principle, better outcomes are obtained when the procedure is performed early on, but this rule does not apply to all cases.

Fisch & Rouleau^4^ reported good and excellent outcomes for two patients submitted to grafting after having facial palsy for 18 and 36 months respectively.

Cazelles et al.^5^ described improved facial motricity in a patient offered grafting after three years of facial palsy.

Salaverry^6^ used grafting to successfully treat two patients with bullet injuries operated one year and four years and two months after the onset of facial palsy.

Patients recovering from grafting three months after surgery reported a sensation of facial strain. This sensation was followed by improved facial symmetry at rest, more clearly observed in the labial sulcus and the mouth. Between three and six months, improvements were seen in the middle third of the face in terms of facial symmetry and tone at rest^8^.

When direct anastomosis was offered, some voluntary motion was seen in the nasolabial area six months after surgery. After six to nine months, facial contractions were stronger. Response of the temporal branch of the facial nerve (frontal area) and of the mandibular branch (lower lip) was limited or absent. Facial motor activity recovery was usually complete between nine and twelve months after surgery^8^. According to a study conducted at Tokyo University, complete recovery may take as long as two to three years^20^.

## CONCLUSION

1. Grafting and anastomosis are adequate solutions to repair sectioned facial nerves, but they never allow for complete facial recovery; synkinesis may occur.
2. In principle, anastomosis is the treatment of choice, but grafting should be preferred when minimal facial nerve stump tensioning is present.
3. Either of the approaches allowed motor recovery rates of 70% on average (72.5% for grafting and 73.3% for anastomosis), with similar quality of recovery.
